# EPS8 variant causes deafness, autosomal recessive 102 (DFNB102) and literature review

**DOI:** 10.1038/s41439-023-00229-w

**Published:** 2023-01-13

**Authors:** Zahra Abbasi, Hossein Jafari Khamirani, Seyed Mohammad Bagher Tabei, Jamal Manoochehri, Mehdi Dianatpour, Seyed Alireza Dastgheib

**Affiliations:** 1 Department of Medical Genetics, Shiraz University of Medical Sciences, Shiraz, Iran; 2 Maternal-fetal Medicine Research Center, Shiraz University of Medical Sciences, Shiraz, Iran; 3 Stem Cells Technology Research Center, Shiraz University of Medical Sciences, Shiraz, Iran

**Keywords:** Neurological disorders, Clinical genetics

## Abstract

Pathogenic variants in the *EPS8* gene result in nonsyndromic hearing loss. This gene encodes the EPS8 protein in cochlear inner hair cells and performs critical roles in stimulating actin polymerization and bundling. Thus far, only four pathogenic variations in *EPS8* have been described. In this study, we report the fifth pathogenic variant in the *EPS8* gene in an Iranian patient with DFNB102. Furthermore, we review literature cases with *EPS8* mutations.

One of the most prevalent sensory impairments, hearing loss (HL), affects approximately 1 in 500 infants. More than 50% of congenital HL cases are due to genetic factors^1,2^. Syndromic HL and nonsyndromic HL (NSHL) are two categories of hereditary HL^3^. Approximately 80% of all hereditary hearing problems are NSHL. These cases are genetically heterogeneous and can have autosomal, sex-linked, or mitochondrial inherited patterns^2^. Thus far, over 120 genes and 6,000 pathogenic mutations in nonsyndromic HL have been identified, and approximately 65% of these genetic factors follow an autosomal recessive (AR) inherited pattern (Hereditary Hearing Loss Homepage; https://hereditaryhearingloss.org accessed Feb 2021). *Epidermal Growth Factor Receptor Pathway Substrate 8 (EPS8)* (MIM#600206) is one of several genes in which mutations cause nonsyndromic hearing loss^4,5^.

*EPS8* is located on 12p12.3 and consists of 21 exons. This gene encodes the EPS8 protein, which is composed of 822 amino acids^5,6^. The EPS8 protein comprises a pleckstrin homology (PH) and SRC homology 3 (SH3) domain^7^.

In the inner ear, specialized mechanoreceptor cells, called hair cells, utilize mechanoelectrical transduction to detect sound. The stereocilia of the auditory cell are arranged in rows of varying heights to produce precise monotone staircase patterns^8^. EPS8 and other proteins situated at the ends of the tallest stereocilia perform critical functions in stimulating actin polymerization and bundling^9,10^. Actin-based movement may be regulated by EPS8, an evolutionarily conserved actin dynamics regulator, by covering the barbed terminal of actin filaments and encouraging actin packing. Furthermore, EPS8 plays a key role in the maturation of inner hair cells, and it is essential for regulating the growth and operation of mammalian auditory hair cells^10^. Mutations in *EPS8* have been identified as causes of deafness and autosomal recessive 102 (DFNB102) (MIM#615974)-related hearing impairment^5,11^. In 2011, Zampini et al. showed that *EPS8* mutations caused profound NSHL in mice^10^. Later, Behlouli et al. (2014) identified the first human case with an *EPS8* mutation, and this individual presented with nonsyndromic profound congenital hearing loss^5^.

Here, we describe an Iranian subject with DFNB102 caused by a pathogenic variant in *EPS8* (NM_004447: c.1259 G > A, p. Trp420Ter). Finally, we review previous cases with *EPS8* mutations.

This article reports an Iranian patient with hearing loss who was born to consanguineous parents (Fig. 1a). The proband (individual V-1) was a 30-year-old female. All the family members were examined properly. Likewise, the subjects’ blood samples were drawn for genetic analysis and supplementary examinations. The patient and parents signed written informed consent forms for the publication of the results. This paper was written according to the CARE Statement^12^.

**Fig. 1:**
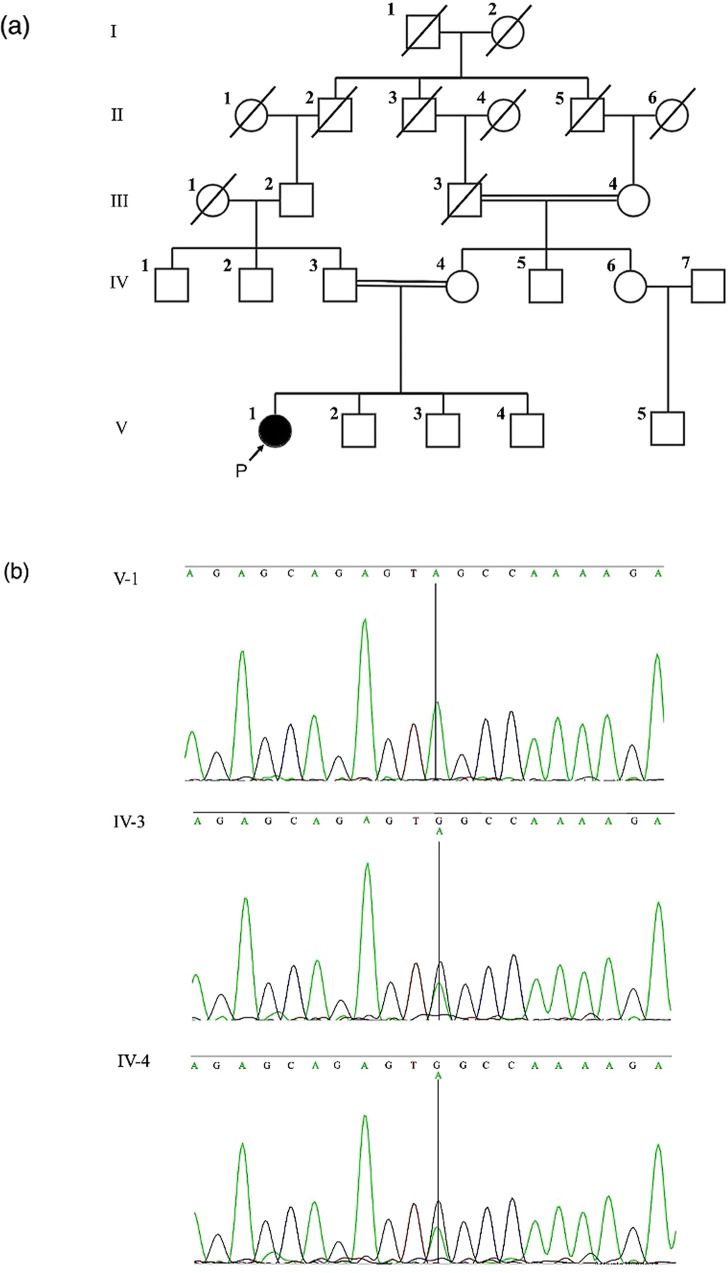
The pedigree and sequencing results of Subject V-1 and her parents. **a** The pedigree shows a recessive inheritance pattern. **b** Sanger sequencing results show a homozygous substitution in the proband and a heterozygous state of this substitution in her parents (Subjects IV-3 and IV-4).

The QIAamp DNA Blood Mini Kit was used to extract genomic DNA from peripheral white blood cells in preparation for sequencing. Following the manufacturer’s instructions, we captured genomic DNA using the SureSelect XT Human All Exon V6 reagent kit (cat. no. 5190-8863; Agilent Technologies, Inc.). Coding DNA samples that were collected were sequenced using an Illumina NovaSeq6000 with 100-bp paired-end sequencing. They were aligned to the human reference genome (hg19) using the Burrows‒Wheeler Aligner^13^. The Genome Analysis Toolkit (GATK) software was used to call single-nucleotide polymorphisms (SNPs). Variants were annotated by ANNOVAR^14,15^. Each variant was assigned to one of five groups based on the ACMG guidelines for the classification of sequence variations: pathogenic, likely pathogenic, variant of unknown significance, likely benign, and benign. The patients’ phenotype was compared to the phenotypic appearance related to the candidate genes. The OMIM database was used to extract the core phenotypic appearance of the mutation (OMIM #615974). Whole-exome sequencing (WES) identified a homozygous pathogenic variant (NM_004447: c.1259 G > A, p. Trp420Ter) in the *EPS8* gene in Subject V-1.

Sanger sequencing verified the presence of this variant. The fragments covering the mutant region were amplified using polymerase chain reaction (PCR). The findings of the Sanger sequencing were analyzed using Chromas Lite v2.01. Oligo Primer Designer V.7 was used to design primers^16^.

The proband was a 30-year-old Iranian female with prelingual, profound, nonsyndromic HL. She was born to consanguineous parents and was delivered naturally by vaginal birth at 40 weeks gestation at a weight of 4,200 g. After birth, her growth and development were normal. Clinical evaluation failed to detect additional symptoms indicating syndromic hearing loss. There were no cochlear-vestibular abnormalities found on her temporal bone CT scan, and she did not have any balance problems. Both parents and the three other siblings did not have signs of hearing impairment.

A homozygous nonsense variant in *EPS8* (NM_004447: c.1259 G > A, p. Trp420Ter) was discovered using whole-exome sequencing of a proband blood sample. An analysis of the segregation of this mutation based on Sanger sequencing confirmed the homozygous variant in the proband and showed that the variant was present in the heterozygous state in both parents (Fig. 1b).

NSHL is genetically heterogeneous, and thus far, numerous genes, such as *EPS8*, have been identified for this type of hearing loss^5^. For instance, the *EPS8* gene mutations that cause autosomal recessive NSHL are known as DFNB102^5,11^.

Thus far, only four pathogenic variations in *EPS8* have been reported in four families. These variants include one nonsense mutation resulting from the substitution of C to T in the coding region (NM_004447: c.88 C > T, p. Glu30Ter), two splicing variants (NM_004447: c.205-8 A > Gp.(?), c.1435-2 A > T, p. His479Cysfs*), and one missense variant resulting from the deletion of an A residue in the coding region (NM_004447: c.115delA, p. Thr39Glnfs*32)^5,11,17,18^. Three of these variations were in a homozygous state (NM_004447: c.88 C > T, c.205-8 A > G, and c.115delA). One had a splice variation that was apparently homozygous due to a 65.9 kb intragenic deletion that covered the variation region (NM_004447: c.1435-2 A > T)^5,11,17,18^. We also identified a fifth pathogenic variation, NM_004447: c.1259 G > A, p. Trp420Ter. This nonsense mutation is predicted to produce either a truncated protein at amino acid position 420 or no protein at all due to nonsense-mediated mRNA decay (NMD)^19^. This variant is classified as “likely pathogenic” based on the PVS1 and PM2 criteria of the ACMG/AMP guidelines. Regarding the clinical characteristics, our patient remarkably resembles the individuals reported previously. The reported affected individuals and our patient manifested profound nonsyndromic hearing loss that presented early in life or at birth (Table 1). They did not have any vestibular malformations or balance problems^11^.

**Table 1:** A review of our patient’s report and all past cases.

Table 1	Family 1	Family 2	Family 3	Family 4	Family 5
Variation	c.88 C > T p.Gln30Ter	c.115delA p.Thr39Glnfs*32	c.205-8 A > G p.(?)	c.1435-2 A > T p.His479Cysfs*	c.1259 G > A p.Trp420Ter
Phenotype	Nonsyndromic HL	Nonsyndromic HL	Nonsyndromic HL	Nonsyndromic HL	Nonsyndromic HL
Descent	Algerian	Tunisian	Pakistani	Chinese	Iranian
Severity of HL	Profound	Mild-to-profound	Severe-to-profound	Profound	Profound
Laterality of HL	Bilateral	N/A	N/A	Bilateral	Bilateral
Zygosity	Homozygous	Homozygous	Homozygous	Apparent homozygosity	Homozygous
Family history	Parental consanguinity	Parental consanguinity	Parental consanguinity	Parental nonconsanguinity	Parental consanguinity
Additional explanations	—	—	—	c.1435-2 A > T and 65.9 kb deletion	—
References	^5^	^18^	^17^	^11^	This study

*N/A* Not available, *HL* Hearing loss.

To conclude, we report the fifth pathogenic mutation in *EPS8* in an Iranian patient affected by profound congenital hearing loss. Her parents and three siblings did not have any hearing impairments. To date, only four patients with pathogenic *EPS8* mutations have been reported. Thus, knowledge of the pathogenic variation of this gene is limited. Reporting the case more rigorously could contribute to diagnosis, genetic counseling leading to more effective supportive care, and even complete care of this disorder employing recently developed technologies.

## HGV Database

The relevant data from this Data Report are hosted at the Human Genome Variation Database at 10.6084/m9.figshare.hgv.3264.

## Data Availability

All data generated or analyzed during this study are included in the final published article.
